# Preliminary Investigation on the Association between Depressive Symptoms and Driving Performance in Heart Failure

**DOI:** 10.3390/geriatrics1010002

**Published:** 2015-12-23

**Authors:** Michael L. Alosco, Marc S. Penn, Mary Beth Spitznagel, Mary Jo Cleveland, Brian R. Ott, John Gunstad

**Affiliations:** 1 Department of Psychological Sciences, Kent State University, Kent, OH 44242, USA; malosco@kent.edu (M.L.A.); mspitzna@kent.edu (M.B.S.); 2 Summa Cardiovascular Institute, Akron, OH 44309, USA; mpenn2@neomed.edu; 3 Center for Senior Health, Summa Health System, Akron, OH 44304, USA; clevelam@summahealth.org; 4 The Alzheimer’s Disease & Memory Disorders Center, Rhode Island Hospital, Warren Alpert Medical School of Brown University, Providence, RI 02903, USA; bott@lifespan.org

**Keywords:** heart failure, driving simulation, cognitive function, depression, aging

## Abstract

Heart failure (HF) patients commit many errors on driving simulation tasks and cognitive dysfunction appears to be one important contributor to impaired driving in HF. Clinical modifiers of cognition may also play a key role. In particular, depression is common in HF patients, linked with cognitive dysfunction, and contributes to reduced driving fitness in non-HF samples. However, the associations among depressive symptoms, cognition, and driving in HF are unclear. Eighteen HF patients completed a validated simulated driving scenario, the Beck Depression Inventory-II (BDI-II), and a cognitive test battery. Partial correlations controlling for demographic and medical confounds showed higher BDI-II score correlated with greater number of collisions, centerline crossings, and % time out of lane. Increased depressive symptoms correlated with lower attention/executive function, and reduced performance in this domain was associated with a greater number of collisions, centerline crossing, and % time out of lane. Depressive symptoms may be related to poorer driving performance in HF, perhaps through association with cognitive dysfunction. However, larger studies with on-road testing are needed to replicate our preliminary findings before recommendations for clinical practice can be made.

## 1. Introduction

Patients with heart failure (HF) exhibit impairments in many instrumental activities of daily living, including management of medications and finances, shopping, and household duties, to name a few [1,2]. Recent work also suggests that some HF patients may be at risk for reduced fitness to drive. For instance, past work from our group shows that HF patients record greater mistakes (e.g., total collisions, stop signs missed, among others) on a driving simulation task relative to healthy controls [3]. These findings are consistent with other work that shows impaired simulated driving in a heterogeneous sample of patients with cardiovascular disease [4].

Poor simulated driving performance in HF has been theorized to largely involve the negative impact of cardiac dysfunction on the brain and subsequent cognitive dysfunction [4,5]. In particular, cognitive impairment in domains important for safe driving (e.g., attention/executive function) is prevalent in HF [4,6] and is correlated with poorer driving simulation performance in this population [3]. Research in other patient populations (e.g., Alzheimer’s disease, Parkinson’s disease) also demonstrates cognitive dysfunction as a key contributor to unsafe driving [7,8,9]. Such findings have resulted in the emergence of cognitive testing as a cornerstone of fitness to drive assessments.

Clinical correlates and modifiers of neurocognition that can be easily assessed by clinicians may also help to identify HF patients at risk for unsafe driving. Although not yet examined, high levels of depressive symptoms can be found in >50% of HF patients [10] and likely contribute to impaired driving in HF. Depressive symptoms are related to poor outcomes in HF (e.g., heightened mortality risk), including greater dependency in instrumental activities of daily living [11] as well as performance reductions on tasks of attention/executive function. The negative effects of depressive symptoms on such driving-related cognitive abilities in HF may translate to impairments in driving fitness and there is some support for this notion in the literature. For instance, past work demonstrates clinically meaningful levels of depressive symptoms are associated with greater likelihood of being involved in a car crash among older adults and 60% of inpatients with major depressive disorder have also been found to be potentially unfit to drive due to mild to moderate impairments in psychomotor function [12,13].

The associations among depressive symptoms, cognitive function, and objectively measured driving abilities in persons with HF is unclear. We sought to expand upon previous work from our group [3,5] and examine the relationship between depressive symptoms and driving simulation performance in older adults with HF. We hypothesized that greater depressive symptoms would negatively impact driving simulation performance, and cognitive dysfunction may offer one possible explanation for this phenomenon.

## 2. Materials and Methods

### 2.1. Participants

The current study was conducted ancillary to an ongoing National Institutes of Health (NIH) study examining neurocognitive function among older adults with HF. HF patients were recruited from outpatient cardiology offices at Summa Hospital in Akron, Ohio. Patients were participating in routine cardiology care for the management and treatment of cardiac dysfunction. For entry into the larger NIH study, all patients were screened for neurological and psychiatric conditions that may influence cognition or functional ability. Specifically, the inclusion criteria were age of 50–85 years, English as a primary language, and a diagnosis of New York Heart Association (NYHA) class II, III, or IV. Individuals were precluded from study entry if they had a history of neurological disorders (e.g., dementia, stroke, multiple sclerosis, *etc.*), head injury with >10 min loss of consciousness, severe psychiatric disorder (e.g., schizophrenia, bipolar disorder), past or current substance abuse/dependence, and stage five chronic kidney disease.

A sample of 21 HF patients was recruited from this larger NIH-funded study to complete a driving simulation task. This sample has also been examined in past studies by our team (for example, see Alosco *et al.*, 2013 [3]). Participants were randomly contacted by phone to participate in the driving study and all participants that expressed interest were then screened for study eligibility. Inclusion criteria required that the participants had a valid driver’s license and were currently driving. Two participants were excluded due to failure to meet inclusion/exclusion criteria (*i.e.*, inactive driver’s license or not currently driving) and one participant withdrew from study procedures (felt uncomfortable during driving simulator). Many participants indeed complained of dizziness or sickness while completing the driving simulation, a common complaint [14], though the current sample persevered through the study. The final sample size included 18 patients with HF with complete driving simulation data. See Table 1 for demographic and medical information of the sample. Of note, the current sample is largely representative of the larger NIH-study from which the participants were recruited (see Alosco *et al.*, 2013 [15]).

**Table 1:** Demographic and Medical Characteristics.

Demographic and Medical Characteristics	
Age, mean (SD)	67.72 (8.56)
Years of Education, mean (SD)	13.78 (2.13)
Female, n (%)	2 (11.1)
BDI-II, mean (SD)	3.00 (4.14)
LVEF, mean (SD)	39.38 (12.45)
Hypertension, n (%)	13 (72.2)
Diabetes, n (%)	7 (38.9)
Myocardial Infarction, n (%)	7 (38.9)
Angiotensin, n (%)	5 (27.8)
ACE inhibitor, n (%)	11 (61.1)
Beta-blocker, n (%)	10 (55.6)
Diuretic, n (%)	4 (22.2)

BDI-II = Beck Depression Inventory-II; LVEF = Left Ventricular Ejection Fraction.

### 2.2. Procedures

The Summa Health System and Kent State University Institutional Review Board (IRB) approved the study procedures and all participants provided written informed consent prior to study enrollment. As part of the larger NIH study’s procedures, a brief neuropsychological test battery was administered and all participants completed the Beck Depression Inventory-II to assess depressive symptoms. Randomly recruited HF participants that completed the above procedures then performed the driving simulation task during a separate study session.

### 2.3. Measures

#### 2.3.1. Driving Simulation

The STISIM Driving Simulator (Build 2.08.03) by Systems Technology Inc. assessed driving ability. It is a computer-based drive simulator software package configured to control: (a) a high fidelity steering wheel with two analog levers for left/right turn indication; (b) an analog pedal set consisting of an analog brake pedal and another pedal for gas; and (c) a 46” High Definition LCD television at an average presentation distance of 4.5 feet. The STISIM driving software was used to devise the driving scenarios that all participants completed. HF participants first completed a practice scenario in order to become familiar with the simulator. The practice scenario is nearly 3 miles long, lasted approximately 15 min, and assessed driving performance in multiple settings, including city, country, and highway environments.

Participants then completed the Kent Multidimensional Assessment Driving Simulation (K-MADS) driving scenario, which is distinct from the practice simulation. All participants were instructed to drive as they normally would on the road and followed a pre-determined course with standard road and traffic signs (e.g., speed and stop signs). The K-MADS is a roughly 7-mile long, 20–25 min driving scenario. It has demonstrated good psychometric properties in adults, including test-retest indices at two weeks that range from *r* = 0.68 to *r* = 0.83 and strong correlations with history of traffic violations in real world driving (*r* = 0.76). The K-MADS measures driving performance in diverse environmental settings, including a quiet suburb, a country road, a small town, and a busy city, each with its own speed limit restrictions and lane configurations. For a full description and review of the K-MADS course, refer to Alosco and colleagues (2012) [16]. The K-MADS automatically tracks and yields several indices reflective of driving performance, including total collisions, number of stop signs missed, number of centerline crossings, number of road excursions, percent of time over the speed limit, and percent of time out of the lane.

#### 2.3.2. Depressive Symptoms

The Beck Depression Inventory-II (BDI-II) [17] operationalized depressive symptoms in the sample. The BDI-II is a well-validated self-report measure of affective and somatic depressive symptoms. Scores range from 0–63 with higher scores reflective of greater depressive symptoms. Specifically, a score between 0–13 signifies minimal depressive symptoms, 14–19 mild depressive symptoms, 20–28, moderate depressive symptoms, and 29–63 is reflective of severe depressive symptoms. The BDI-II served as a continuous variable in all primary analyses and the above-described cut-offs were only used for descriptive purposes.

#### 2.3.3. Cognitive Function

A cognitive test battery was administered to all participants to assess attention/executive function and memory. Specifically, Trail Making Test A and B [18] and Digit Symbol Coding [19] operationalized attention/executive function in this domain. The California Verbal Learning Test-Second Edition (CVLT-II) examined memory abilities. CVLT-II short and long delay free recall were used as the dependent variables. All neuropsychological tests in this study demonstrate strong psychometric properties, and Trail Making Tests in particular are widely used in clinical practice to assess cognitive function and fitness to drive [20,21].

#### 2.3.4. Demographic and Medical History

Demographic and medical history was ascertained via participant self-report and medical chart review. Participants first completed a medical history questionnaire to self-report demographic variables (e.g., age, education, *etc.*), as well as diagnostic history of medical conditions such as hypertension, myocardial infarction, and type 2 diabetes mellitus. A trained research assistant then performed a medical chart review to corroborate participants’ self-reported information. As part of routine clinical care, all HF patients underwent an echocardiogram during their outpatient cardiology appointments prior to entry into the larger NIH study of which the current sample was recruited. Medical record review was used to obtain the left ventricular ejection fraction (LVEF) of each participant derived from that echocardiogram.

### 2.4. Statistical Analyses

Simple mean imputation was used to estimate LVEF for two cases with missing data on this variable. Neuropsychological measures of attention/executive function and memory were converted to T-scores using clinical normative data (e.g., Halstead-Reitan and specific test developer manuals) that takes into account age and gender for tasks that assessed memory. A T-score <35 represents 1.5 SD below the mean of the normative population and was used to operationalize impaired test performance. To limit the number of analyses, attention/executive function and memory composite scores were created that consisted of the mean T-scores of the measures that comprise their respective domain.

All variables were normally distributed. Partial correlation analyses examined the associations among BDI-II scores, cognitive function, and the driving simulation indices. To preserve statistical power, only variables that have been shown to influence driving ability were included as covariates and included age, type 2 diabetes mellitus, and diagnostic history of myocardial infarction. HF severity was also included as a control variable in order to account for the variance of disease severity—that is, we sought to determine whether the effects of depressive symptoms on driving ability are independent of disease status. Independent samples *t*-tests and bivariate correlations were also conducted to explore the relationship among other potential demographic (e.g., education, sex) and medical (e.g., hypertension) confounds with driving simulation performance. Variables that demonstrated significant associations with driving indices were then included as covariates. Due to the small sample size and the exploratory nature of this study, Bonferroni corrections were not made for multiple comparisons.

## 3. Results

### 3.1. Driving Simulation Errors

Refer to Table 2 for a full summary of driving simulation performance in the sample. Consistent with past work, HF patients committed many errors on the driving simulation task. Nearly all participants recorded at least 1 collision and more than 60% (*n* = 11) had ≥2 collisions. Off-road excursions (mean (SD) = 4.83 (6.39)) were also common and more than 50% (*n* = 10) missed at least one stop sign. In terms of demographic variables, bivariate correlations showed older age was associated with a greater number of stop signs missed (*r*(degrees of freedom (df) = 16) = 0.51, *p* = 0.03) and off road excursions (*r*(df = 16) = 0.54, *p* = 0.02); neither sex nor education were associated with any of the driving simulation indices (*p* > 0.05 for all). Diagnostic history of hypertension also did not emerge as a significant contributor to driving simulation (*p* > 0.05 for all simulation indices). Given the null effects between sex, education, and hypertension with driving simulation performance, these variables were not included in analyses that examined driving simulation indices.

**Table 2:** Driving Simulation Performance.

	Mean (SD)	Range
Total Collisions	2.00 (1.28)	0–5
Number of Stop Signs Missed	0.78 (0.81)	0–2
Number of Centerline Crossings	5.83 (3.96)	1–12
Number of Road Excursions	4.83 (6.39)	0–23
% Over Speed Limit	9.06 (8.57)	0–29.88
% Out of Lane	4.75 (3.16)	0.95–11.88

### 3.2. Depressive Symptoms and Driving Simulation Performance

The average BDI-II score of the sample was 3.00 (SD = 4.14) with scores ranging from 0 to 15. Nearly all participants fell in the range reflective of minimal depressive symptoms (*i.e.*, 0 to 13), and eight subjects reported no symptoms of depression, with the exception of one participant that exhibited moderate levels of depressive symptoms. BDI-II scores were not associated with age, sex, education, LVEF, diagnostic history of hypertension, type 2 diabetes mellitus, or myocardial infarction (*p* > 0.05 for all).

Higher BDI-II scores emerged as a significant correlate of worse driving simulation performance. Partial correlations that adjusted for age, LVEF, diagnostic history of type 2 diabetes mellitus, and myocardial infarction revealed a significant association between the BDI-II with the following driving simulation indices: total collisions (*r*(df = 12) = 0.56, *p* = 0.04), number of centerline crossings (*r*(df = 12) = 0.68, *p* = 0.01), and percent of time spent out of the lane (*r*(df= 12) = 0.70, *p* = 0.01). There were no other significant associations with the remaining driving simulation indices (See Table 3).

**Table 3:** BDI-II and Driving Simulation Partial Correlations.

Driving Simulation Indices	BDI-II	*p*-Value
Total Collisions	0.56	0.04
Stop Signs Missed	0.44	0.11
Centerline Crossings	0.68	0.01
Road Excursions	−0.12	0.69
% Time Over Speed Limit	0.40	0.16
% Time Out of Lane	0.70	0.01

*Note*: BDI-II = Beck Depression Inventory-II; Analyses adjusted for age, left ventricular ejection fraction, type 2 diabetes mellitus, and history of myocardial infarction.

### 3.3. Depressive Symptoms and Driving Simulation: The Role of Cognitive Function

Cognitive impairment was common in this sample, particularly in attention/executive function. Specifically, 11.1% (*n* = 2) exhibited impaired performance (*i.e.*, T-score < 35) on the Trail Making Test A, and 22.2% (*n* = 4) demonstrated impairments on the Trail Making Test B; no participants scored below a T-score of 35 on the Digit Symbol Coding task. In terms of memory, 11.1% (*n* = 2) of the sample was impaired on the CVLT-II long delay free recall, but there were no impairments found on the CVLT-II short delay free recall. The BDI-II demonstrated a significant association with the attention/executive function composite score (*r*(df = 11) = −0.59, *p* = 0.03), even after controlling for variables that influence cognitive function in HF such as age, LVEF, hypertension, type 2 diabetes mellitus, and myocardial infarction. Greater depressive symptoms were associated with worse cognitive function in this domain. This pattern did not emerge for memory (*p* > 0.05).

The attention/executive function composite correlated with worse simulated driving performance on the same indices impacted by depressive symptoms, suggesting cognitive dysfunction may be one possible mechanism by which depressive symptoms affect driving abilities. Specifically, worse attention/executive function correlated with a greater number of total collisions (*r*(df = 12) = −0.63, *p* = 0.02), a higher number of centerline crossings (*r*(df = 12) = −0.68, *p* = 0.01), and a greater time spent out of the lane (*r*(df = 12) = −0.65, *p* = 0.01). However, there was no relationship between memory and driving simulation performance (*p* > 0.05), see Table 4. For those driving indices that demonstrated a significant effect with the BDI-II (total collisions, centerline crossings, and percent time out of the lane), exploratory partial correlations analyses showed this effect diminished after controlling for attention/executive function (in addition to the other described covariates), supporting cognitive function as a possible mechanism.

**Table 4:** Partial Correlations Examinging Cognitive Function and Driving Simulation Performance.

Driving Simulation Indices	Attention/Executive Function (*p*-value)	Memory (*p*-value)
Total Collisions	−0.63 (0.02)	0.36 (0.21)
Stop Signs Missed	−0.25 (0.39)	0.30 (0.30)
Centerline Crossings	−0.68 (0.01)	0.05 (0.87)
Road Excursions	−0.11 (0.71)	−0.30 (0.30)
% Time Over Speed Limit	0.02 (0.94)	0.35 (0.23)
% Time Out of Lane	−0.65 (0.01)	−0.04 (0.91)

Partial Correlations are adjusted for age, left ventricular ejection fraction, type 2 diabetes mellitus, and history of myocardial infarction.

## 4. Discussion

This sample of persons with HF committed frequent errors on a simulated driving task. Cognitive dysfunction appears to in part underpin impaired driving in HF, although clinical and health-related factors may also play a key role. Depressive symptoms can be found in >50% of patients with HF, and the current preliminary study among a small sample of HF patients suggests that depressive symptoms may contribute to driving simulation performance in this population.

Increased depressive symptoms emerged as a significant correlate of greater number of total collisions, centerline crossings, and more time spent out of the lane on a driving simulation scenario. These findings are consistent with work in older adults that shows depressive symptoms are associated with heightened risk for vehicle crash involvement [12]. One possible explanation for these findings may involve the known association between depressive symptoms and suppressed mental abilities, particularly attention/executive dysfunction and psychomotor slowing. As an example, Brunnauer and colleagues [13] found that 16% of inpatients with major depressive disorder were deemed unfit to drive due to severe impairments in psychomotor function, and driving abilities were also questionable among an additional 60% of the inpatients that exhibited mild to moderate psychomotor speed impairments. We also found a significant association between depressive symptoms and higher-ordered cognitive tasks critical for optimal driving performance, including measures that tap into psychomotor speed, multi-tasking, and visual attention. Impaired performances in these cognitive functions correlated with the same driving simulation indices affected by depressive symptoms (*i.e.*, total collisions, centerline crossings, and time spent out of the lane) in this patient sample and also contribute to poorer driving skills in other patient populations (e.g., stroke, Parkinson’s disease) [22,23]. Intact attention/executive functions are necessary to perform routine vehicle operations such as switching lanes, quick and safe decision-making, processing incoming traffic information, monitoring and controlling speed, among other driving tasks that require a high cognitive load.

Depressive symptoms contribute to poor outcomes in HF (e.g., repeated hospitalizations) [24] and is thus a key therapeutic target in this population. Depressive symptoms can be alleviated in patients with HF through psychotropic, psychotherapeutic, and/or behavioral (e.g., cardiac rehabilitation) interventions [25], and such improvements appear to promote functional independence in cardiac patients, including better adherence to medication regimens and other self-care behaviors [26]. Although speculative at this time, it is possible that reductions in depressive symptoms may also translate to better driving skills. There is some support for this in the literature that shows that antidepressant medications improve performance on standardized road and traffic safety assessments of driving-related mental functions in older adults (e.g., visual perception, reaction time, selective attention, vigilance, and stress and tolerance) [13]. Driving cessation can produce significant practical and emotional problems and future work is needed to examine potential interventions that may preserve driving skills in HF, including the role of psychological treatment.

The current findings are limited in several ways. First, the small sample size, lack of an age-matched control group, and the homogeneity of the sample limits generalizability and our findings should be considered preliminary. Future case-controlled studies with larger samples are needed to replicate our findings and also implement statistical modeling to provide a more robust examination of the interrelations among depressive symptoms, cognition, and driving performance, and account for potential confounds that may have influenced our results (e.g., driving experience, HF duration and severity). The cross-sectional nature of the current study limits interpretation of directionality among depressive symptoms, cognitive function, and driving simulation performance and larger longitudinal studies that implement healthy controls and comparison groups are needed to confirm and clarify these relationships. Larger case-controlled studies that employ on-road testing are also needed to determine whether depressive symptoms and cognitive function screening in the clinical care of HF patients may help clinicians to detect possible driving safety concerns.

We also implemented driving simulation to operationalize driving fitness, which is a safe, inexpensive, and face valid method; however, driving simulators lack ecological validity, can be confounded by motion sickness, and do not assess on-road driving indices (e.g., crash statistics). Future studies that employ on-road testing and/or thorough examinations of driving records (e.g., traffic tickets, crash history) would help to increase our understanding of the influence of depressive symptoms on driving in HF. Non-cognitive mechanisms by which depression may influence driving skills were also not examined in this study. For instance, depressive symptoms may adversely impact on-road decision-making due to the emotional substrates that comprise this psychological disorder, such as reduced self-esteem, low confidence, and rumination, among others. It is also important to consider possible behavioral reasons for the inverse association between depressive symptoms and neuropsychological testing, including response biases and reduced motivation to perform at an optimal level. While such behavioral maladaptation may misrepresent patient’s actual cognitive abilities, they also likely translate to real-world function and affect behind-the-wheel performance. The current sample exhibited mild levels of depressive symptoms, with many reporting none, perhaps due to the relatively mild levels of cardiac function that may, in part, stem from the young age of the sample; although, the burden of HF rests in the ≥65 age group, which is consistent with the average age of 67 in this sample [27,28]. While the small subset of HF participants with more meaningful levels of depression may have accounted for our findings, it is plausible that mild symptoms of depression may influence driving performance. Overall, in order to better clarify the role of depressive symptoms in driving performance in HF, larger, more disease-diverse samples of HF patients are needed; such work will also clarify the differential effects of the various cardiac subtypes on depressive symptoms and driving.

## 5. Conclusions

In conclusion, the current study provides preliminary evidence for depressive symptoms as a possible risk factor for unsafe driving in HF, possibly due to its association with cognitive dysfunction. However, larger longitudinal studies that employ on-road testing are needed to replicate our findings before clinical recommendations can be made.
